# Author Correction: Films of Bacteria at Interfaces (FBI): Remodeling of Fluid Interfaces by *Pseudomonas aeruginosa*

**DOI:** 10.1038/s41598-019-54303-x

**Published:** 2019-11-25

**Authors:** Tagbo H. R. Niepa, Liana Vaccari, Robert L. Leheny, Mark Goulian, Daeyeon Lee, Kathleen J. Stebe

**Affiliations:** 1 Department of Chemical and Biomolecular Engineering, University of Pennsylvania, Philadelphia, PA 19104 USA; 2 Department of Physics and Astronomy, Johns Hopkins University, Baltimore, MD 21218 USA; 3 Department of Biology, University of Pennsylvania, Philadelphia, PA 19104 USA

Correction to: *Scientific Reports* 10.1038/s41598-017-17721-3, published online 19 December 2017

The original HTML version of this Article contained an error in the order of the five Supplementary Video files. This error has now been corrected in the HTML; the PDF version of the paper was correct from the time of publication.

