# Supplementary material for: Films of Bacteria at Interfaces (FBI): Remodeling of Fluid Interfaces by Pseudomonas aeruginosa
Source: Sci Rep. 2017 Dec 19;7:17864. doi: 10.1038/s41598-017-17721-3 (PMC5736630; doi:10.1038/s41598-017-17721-3)
Supplement: Supplementary file 1 — Supplementary information [file 41598_2017_17721_MOESM1_ESM.pdf]

Supplementary information

for

Films of Bacteria at Interfaces (FBI): Remodeling of Fluid Interfaces by *Pseudomonas aeruginosa*.

Tagbo H. R. Niepa[1#], Liana Vaccari[1#], Robert L. Leheny[2], Mark Goulian[3], Daeyeon Lee*[1] and Kathleen J. Stebe*[1]

[1]Department of Chemical and Biomolecular Engineering, University of Pennsylvania, Philadelphia, PA 19104, USA
[2]Department of Physics and Astronomy, Johns Hopkins University, Baltimore, MD 21218, USA
[3]Department of Biology, University of Pennsylvania, Philadelphia, PA 19104, USA,

#Equal Contribution Authors

*Corresponding author:
Email: kstebe@seas.upenn.edu, daeyeon@seas.upenn.edu.

We include in this supplementary information five representative emulsion and drop compression experimental videos, referred to in the main text. Furthermore, we discuss how particle tracking data is corrected for drift, and how the exponent of the power law for the mean square displacement, reported in the main text, is determined. We also address issues associated with the microbe itself. We provide a discussion of a phenotypic phenomenon caused by genetic drift, and the viability of bacteria at oil-water interfaces. We also address interfacial coverage by bacteria of a typical particle tracking experiment. Lastly, as is customary in work with genetic analysis, we include the complete results of RNA sequencing of *P. aeruginosa* PAO1 and PA14.

Videos

Supplementary Video 1 – Aging of emulsion: Droplets in the sample containing PA14 cells are short lived, and the oil and water phases separated rapidly (5 min). However, droplets formed in the presence of PAO1 are highly stable, presumably because of more efficient trapping and restructuring of the interface, allowing oil droplets surrounded by water to remain intact up to 10 days.

Supplementary Video 2 – PAO1 wildtype pendant drop compression: The drops in contact with the PAO1 suspension show evidence of a wrinkled "bag"-like structure upon compression, suggesting that the FBI covering the interface is a thin solid film with finite bending modulus.

Supplementary Video 3 – PA14 wildtype pendant drop compression: Hexadecane drops aged in the presence of a PA14 suspension shows no structure formation. Rather, upon compression, the drop shrinks like a typical liquid drop.

Supplementary Video 4 – PA14Δ*pilC* pendant drop compression: Upon withdrawal of oil from the pendant drop aged in contact with PA14Δ*pilC*, wrinkles form, suggesting a finite bending modulus to this layer. Interestingly, films of these de-piliated microbes also show evidence of microbes leaving the interfacial region upon compression. Some bacteria escape from the film formed by the PA14Δ*pilC* mutants upon drop compression from the apex of the drop, indicating that some population is only weakly cohered to the film.

Supplementary Video 5 – PAO1Δ*alkB2* pendant drop compression: A hexadecane drop aged in the presence of PAO1Δ*alkB2* showed no signs of wrinkling under compression, and therefore no signs of an elastic film at the surface.

1) Mean Squared Displacement of particles and determination of power law exponent.

These figures demonstrate the evaluation of particle motion at the interface in the presence of bacteria. At each interface age, the drift subtracted individual mean squared displacement (iMSD) is evaluated for every particle. These iMSDs are averaged to achieve the ensemble mean

squared displacement (eMSD) for that interface age. We evaluate these over a lagtime of 100 frames or 1.67 seconds. We chose this lagtime because fewer particles are visible at long lagtimes owing to drift. There are also fewer particles sampled on interfaces that become elastic at long interface ages owing to a lack of drift, which precludes particles from moving into the field of view. Even with this reduced number of particles, the behavior in the elastic films is quite uniform, as indicated by the narrow distribution of iMSDs. Each line in Fig S1c is the eMSD for the particles at a given interface age, and each marker in Fig S1d corresponds to *n*, the slope of this eMSD at that age. The parameter, *d*, root mean squared displacement, is evaluated at 1.67 seconds.

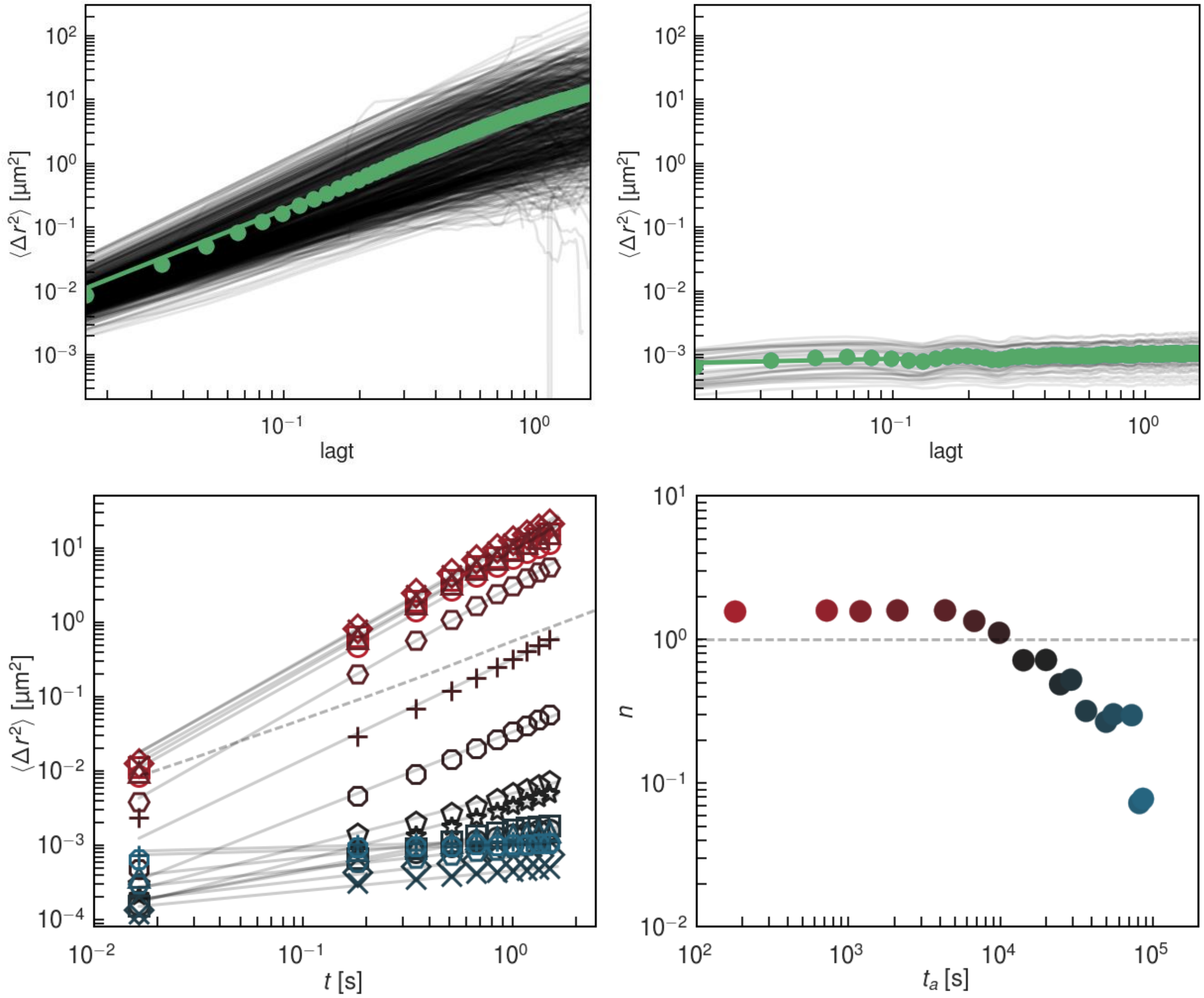

Figure S1 Example analysis of particles that gives rise to the *n* and *d* data described in the main text. **(a)** and **(b)** Mean square displacements at interface age, $t_a = 60$ s and $t_a = 86460$ s, respectively. Thin black lines are each the individual mean squared displacement of a particle. The green markers are the ensemble average of these MSDs, and the green line is the best fit to the ensemble data from which the slope, *n*, is evaluated. **(c)** eMSDs, where each color corresponds to the interface age represented in **(d)**, with gray lines to guide the eye and shows the evolution of the interfacial behavior over time, dashed line representing the displacement

MSD of particles at a clean interface, which has a slope of 1. **(d)** *n*, slope of the eMSDs at each interface age, again with $n = 1$ shown as a reference of particles at a clean interface.

2) Elaboration of drift subtraction

Drift subtraction has been performed by calculating the mean displacement frame by frame in the x and y directions for the population of particles and subtracting this average velocity before calculating the MSD. This correction greatly reduces but does not fully eliminate contributions from drift, since one can have a gradient in the drift velocity across the field of view. This effect is visible at large lagtimes, therefore we restrict our analysis to smaller lagtimes where it is negligible. The lagtime for which we report our data (1.67s) is significantly smaller than the lagtimes that suffer from the effects of drift. This correction is similar to that discussed in Samaniuk & Vermant, 2014, and has been adopted by careful practitioners of this method, including in our prior work exploiting interfacial probes in Lee et al., Langmuir **25**, 7976-7982, 2009 and in Crocker and Grier, JCIS **179**, 298-310, 1996.

3) Effect of P1 and P2 phenotypic variations in PAO1 on the formation of elastic films at hexadecane-water interfaces. Sublines of PAO1 isolates are known to undergo microevolution through genetics changes in the MexEF-OprN efflux system[1] that lead to phenotypic variations. Here, we confirm the formation of an elastic FBI from various sublines of PAO1, including PAO1 VID1, PAO1-VIE6, MPAO1, and PAO1 (o) originally used for this study. The PAO1 knockouts studied in this work were derived from MPAO1 from the University of Washington[2], which displays the P1 and P2 phenotypes, the latter being characterized by a higher sensitivity to chloramphenicol (Cm) and increased virulence. To confirm that the elastic film formation was independent of these variations, analyses were performed using the MPAO1 mutants expressing the two phenotypes. Growth curves were recorded for cells grown in the presence or absence of 30 µg/mL Cm for 8 h (Supplementary Fig. 1.) All strains tolerate Cm30, except the MPAO1 mutants of Δ*PelA,* Δ*pslD,* Δ*rhlA,* and Δ*alkB2* that exhibited the P2 phenotype. When exposed to the hexadecane-water interface, each PAO1 knockout, barring the mutant of Δ*alkB2*, formed an elastic FBI similar to the wildtype. Mutants including Δ*PelA*, Δ*pslD*, Δ*rhlA* form an elastic film as shown in Fig. S1b and Fig. 3 (in the main text). All the wild type strains used in the study also form the elastic films under interfacial confinement. Elastic moduli for these strains: PAO1 VID1 11.8 µPa-m, PAO1-VIE6, 4.9±1.3 µPa-m, MPAO1, 3.8 µPa-m, and PAO1 (o), 2.6 µPa-m. Our results show that the formation of an elastic films is independent of phenotypic variation in PAO1 cells.

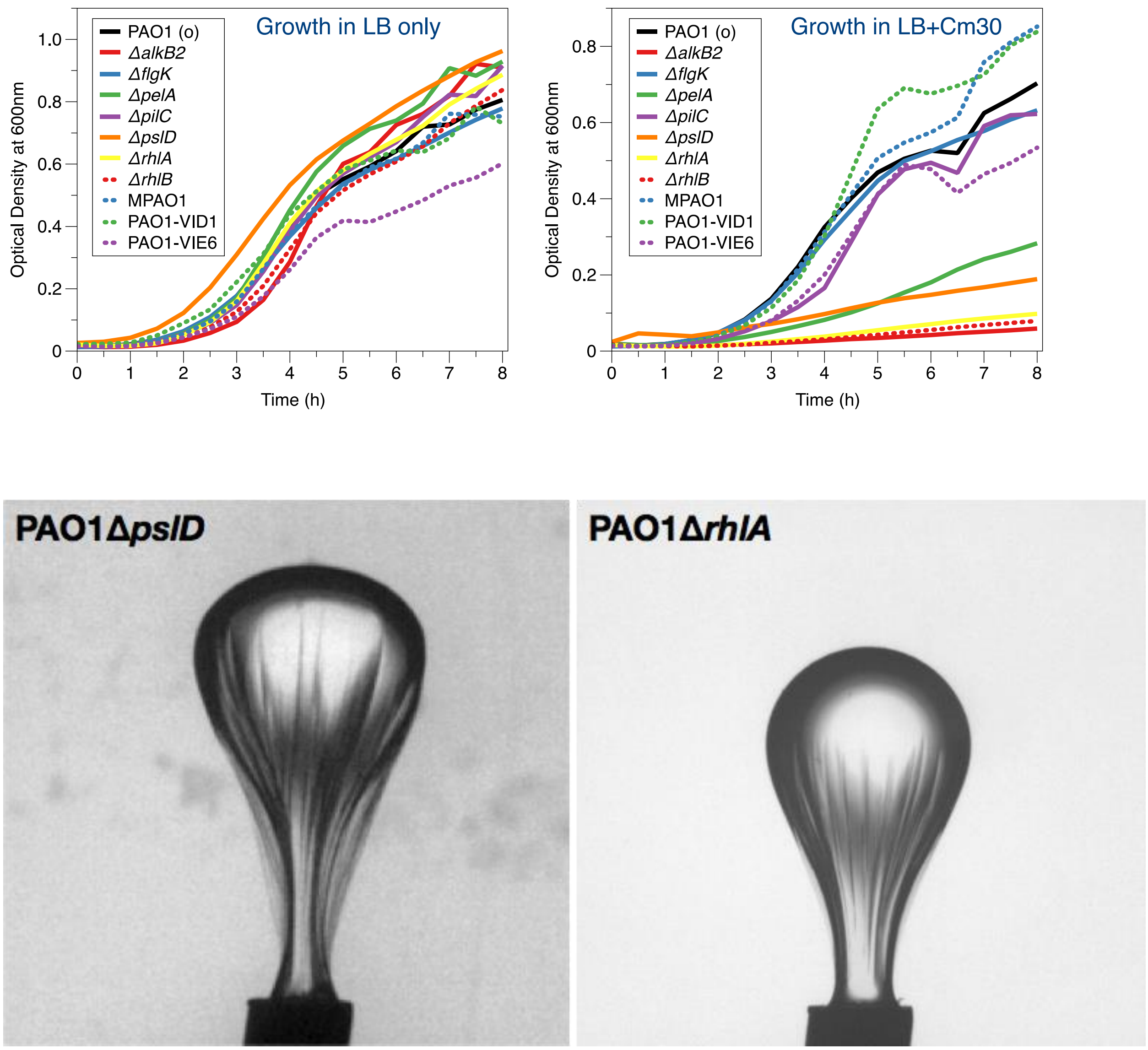

Figure S3 (a) Growth curves of *P. aeruginosa* strains in the absence and the presence of 30μg/mL chloramphenicol (Cm30), showing that mutants including Δ*PelA*, Δ*pslD*, Δ*rhlA*, and Δ*alkB2* exhibit P2 phenotype. **(b)** Pendant drop elastometry on a hexadecane droplet held at the tip of an inverted needle in a bacteria suspension of PAO1Δ*pslD* and MPAO1Δ*rhlA* for 24 h. Both strains exhibit the P2 phenotype and form an elastic film under interfacial confinement.

4) Cell viability at fluid interfaces. To determine if stationary PAO1 and PA14 cells adhering to a hexadecane-water interface for 1 h remained viable, Live/Dead staining assay was performed. Emulsions of hexadecane generated through the mixing of a bacterial suspension with hexadecane were stained using the Filmtracer LIVE/DEAD Biofilm Viability Kit (Thermo Fisher Scientific). The following images showed that both PAO1 and PA14 are viable at the hexadecane-water interfaces, although some dead cells were observed at PA14-laden interfaces.

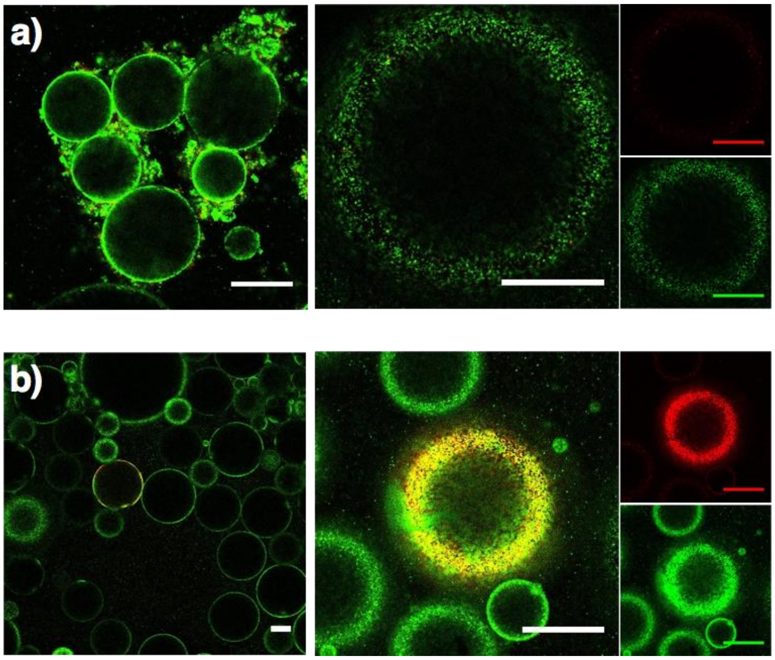

Figure S3 – Live/dead imaging of bacteria at fluid interfaces. Wild type of (a) PAO1 and (b) PA14 cells were confined at hexadecane-water interfaces for 1 h and stained to assess cell viability. Dead cells are stained in red via propidium iodide, while all cells are stained in green via membrane permeable dye, Syto 9. The PAO1 cells remained viable at fluid interfaces. Also, most PA14 cells at the interface remain viable although we observed a small number of droplets with dead cells (scale bar: 100 μm).

5) Changes in surface density of bacteria over time. In some cases, we could extract a number count for the cells on the interface from the video images; however data are not detailed enough to extract the orientation of cells at the interface. To consider these number counts in terms of surface densities, reference possible area/microbes are calculated. If a bacterium locates with its body in the plane of the interface, but with flagella playing no role in the interfacial area, the area per microbe on the interface would be the body cross section $A_B = 0.71\ \mu m^2$ (Wilson, *et al.* AEM **70**, 2004, 5847-5852). If, however, the flagella in the interface plane create a zone of excluded area directly behind the cell, a reference area/microbe could be given by the product of the cell body width of 1 μm and the sum of cell body and flagella length of 5 μm, resulting in an estimated interfacial area of the cell body and flagellum $A_{BF} = 5\ \mu m^2$. A third area per cell of interest is an area per cell explored if the cell and flagella, of length 5 μm, interact anywhere within a circle of that radius; $A_{Encounter} = 78.5\ \mu m^2$.
In a typical field of view, we image a domain of ~150 μm x 200 μm; if 400 cells were in this domain, the average area per cell would correspond to the of $A_{Encounter}$; 6240 cells would correspond to A_{B+F}, and 44,000 cells would correspond to $A_{B.}$ The number counts for representative cases are reported in Table S1. The average area per cell is several times larger than the encounter area, and range from 0.1-0.3 of the A_{BT}.

Table S1: Cell counts for three bacteria strains at five interface ages.

Approximate interface age (hr)	PAO1 WT	PAO1 ΔalkB2	PA14 WT
0	814	1208	1891
0.5	1360	800	2463
1	1503	1239	2633
8	1806	1222	1964
24	1269	1626	1931

6) Transcriptional profile of cells associated with active layers and elastic films. *P. aeruginosa* PAO1 at the hexadecane-water interface formed an elastic film, while PA14 was unable to do so. The variation in the response of PAO1 and PA14 after exposure at the oil-water interfaces was not associated with cellular features involved in the formation of their biofilms. To further understand how the confinement at the hexadecane-water interfaces affected the cells, RNA sequencing was performed on PAO1 and PA14 cells in the absence and presence of interfacial confinements, and the transcriptional profiles of the cells were compared. The following figures and tables present the functions affected by the exposure to the hexadecane-water interfaces for PAO1 and PA14.

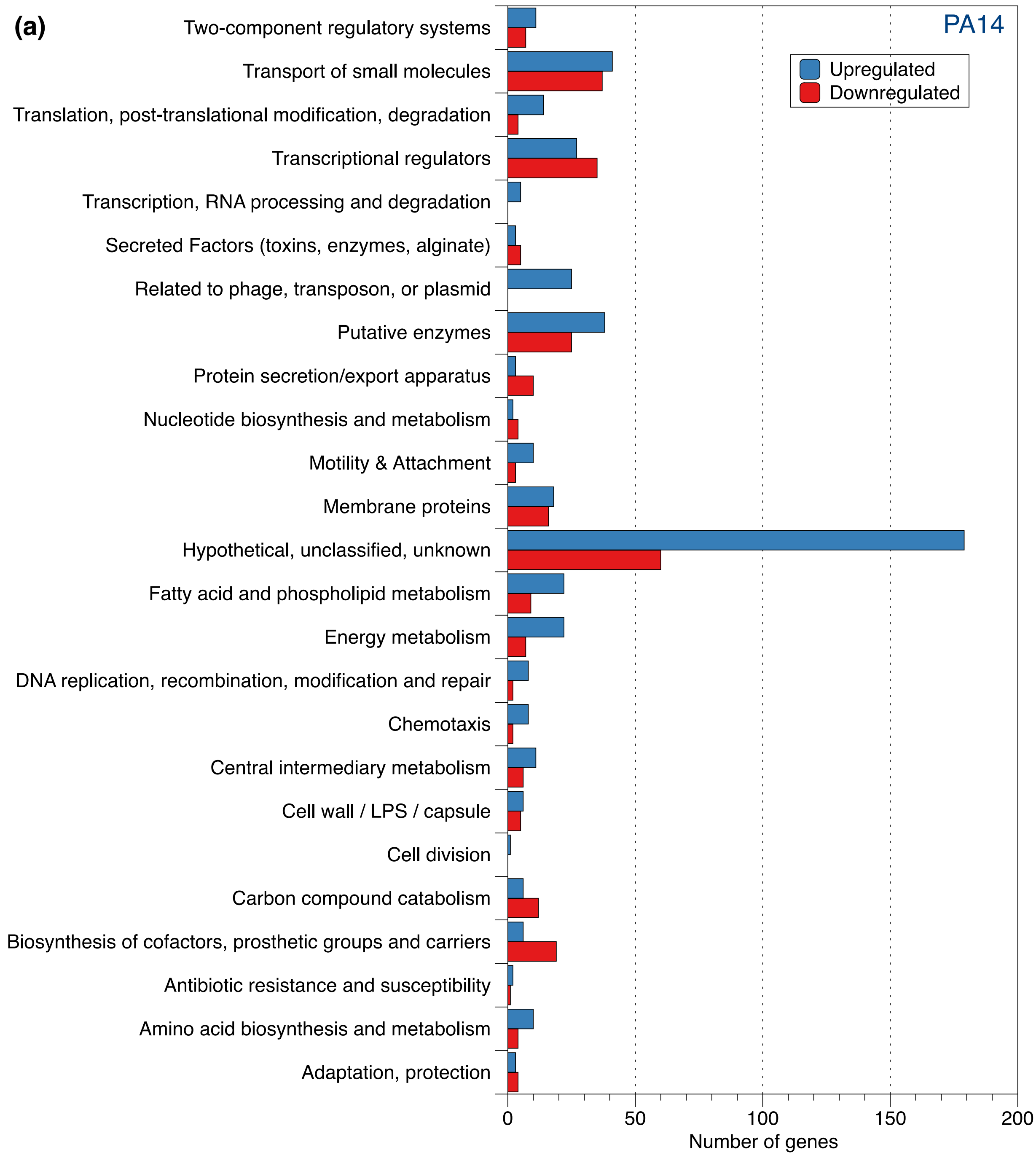

(a)
PA14
Upregulated
Downregulated
Two-component regulatory systems
Transport of small molecules
Translation, post-translational modification, degradation
Transcriptional regulators
Transcription, RNA processing and degradation
Secreted Factors (toxins, enzymes, alginate)
Related to phage, transposon, or plasmid
Putative enzymes
Protein secretion/export apparatus
Nucleotide biosynthesis and metabolism
Motility & Attachment
Membrane proteins
Hypothetical, unclassified, unknown
Fatty acid and phospholipid metabolism
Energy metabolism
DNA replication, recombination, modification and repair
Chemotaxis
Central intermediary metabolism
Cell wall / LPS / capsule
Cell division
Carbon compound catabolism
Biosynthesis of cofactors, prosthetic groups and carriers
Antibiotic resistance and susceptibility
Amino acid biosynthesis and metabolism
Adaptation, protection
0
50
100
150
200
Number of genes

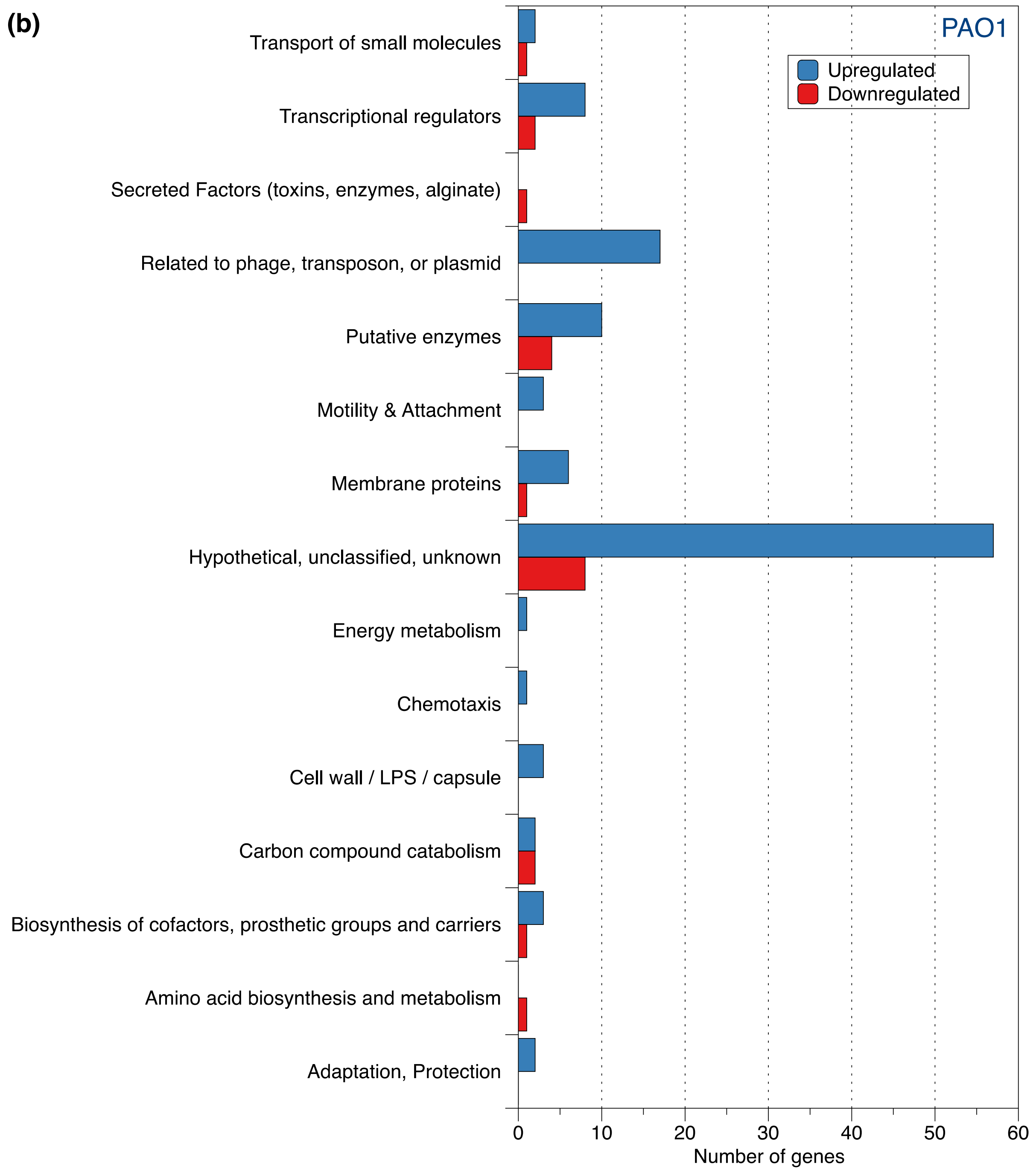

Figure S6: Transcriptional profile of **(a)** PA14 and **(b)** PAO1 after confinement at hexadecane-water interface for 1h. The number of genes upregulated and downregulated is presented for the genes functions.

Table S2: List of PA14 genes upregulated during confinement at hexadecane-water interface for 1h.

Gene		Fold Change	p value
	Adaptation, protection		
PA14_72760	beta-lactamase	2.17	0.0000
PA14_12300	hypothetical protein	2.47	0.0000
PA14_41880	universal stress protein	2.51	0.0000
	Amino acid biosynthesis and metabolism		
PA14_33030	L-serine dehydratase	2.04	0.0000
PA14_65110	biosynthetic alanine racemase	2.09	0.0000
PA14_46070	guanidinobutyrase	2.34	0.0000
PA14_08360	indole-3-glycerol-phosphate synthase	2.47	0.0000
PA14_41830	phosphoserine phosphatase	2.72	0.0000
PA14_52660	succinylarginine dihydrolase	2.77	0.0000
PA14_08350	anthranilate phosphoribosyltransferase	2.86	0.0000
PA14_05070	methionine biosynthesis protein	3.46	0.0000
PA14_63110	S-adenosylmethionine decarboxylase	3.53	0.0000
PA14_46860	lysine decarboxylase	5.76	0.0000
	Antibiotic resistance and susceptibility		
PA14_32390	RND multidrug efflux transporter MexF	2.04	0.0000
PA14_36280	antibiotic biosynthesis monooxygenase	2.22	0.0000
	Biosynthesis of cofactors, prosthetic groups and carriers		
PA14_27850	7-cyano-7-deazaguanine reductase	2.16	0.0000
PA14_04050	proline hydroxylase	2.38	0.0000
PA14_07600	2-amino-4-hydroxy-6- hydroxymethyldihydropteridine pyrophosphokinase	2.43	0.0000
PA14_38820	pyrroloquinoline quinone biosynthesis protein PqqB	2.45	0.0000
PA14_38825	coenzyme PQQ synthesis protein PqqA	2.53	0.0000
PA14_21640	short chain dehydrogenase	9.01	0.0000
	Carbon compound catabolism		
PA14_71490	sarcosine oxidase delta subunit	2.11	0.0026
PA14_30810	alkane-1 monooxygenase	2.13	0.0000
PA14_32240	catechol 1"	2.28	0.0003
PA14_32230	muconolactone delta-isomerase	2.57	0.0027
PA14_32220	muconate cycloisomerase I	5.32	0.0002
PA14_19700	Aldolase	8.93	0.0000
	Cell division		
PA14_25610	Maf-like protein	2.08	0.0000
	Cell wall / LPS / capsule		
PA14_69660	lipopeptide LppL	2.01	0.0001
PA14_61650	Lipid A 3-O-deacylase	2.03	0.0000

PA14_00120	lipid A biosynthesis lauroyl acyltransferase	2.05	0.0000
PA14_38360	nucleotide sugar dehydrogenase	2.48	0.0000
PA14_63030	outer membrane lipoprotein OmlA precursor	2.84	0.0000
PA14_12280	apolipoprotein N-acyltransferase	3.11	0.0000
	Central intermediary metabolism		
PA14_08390	S-adenosylmethionine decarboxylase	2.01	0.0000
PA14_38350	UTP-glucose-1-phosphate uridylyltransferase	2.06	0.0000
PA14_64950	hypothetical protein	2.15	0.0000
PA14_41840	phosphoadenosine phosphosulfate reductase	2.21	0.0000
PA14_36890	Metallothionein	2.21	0.0000
PA14_48620	clavaminic acid synthetase	2.28	0.0000
PA14_19470	malate:quinone oxidoreductase	2.46	0.0000
PA14_41530	assimilatory nitrite reductase large subunit	2.51	0.0000
PA14_11770	ethanolamine ammonia-lyase large subunit	2.81	0.0000
PA14_41540	assimilatory nitrite reductase small subunit	3.29	0.0000
PA14_64520	Bacterioferritin	3.34	0.0000
	Chemotaxis		
PA14_30820	methyl-accepting chemotaxis transducer	2.10	0.0000
PA14_29800	chemotaxis transducer	2.12	0.0000
PA14_43710	methyl-accepting chemotaxis transducer	2.44	0.0000
PA14_55960	chemotactic transducer PctC	2.52	0.0007
PA14_39560	chemotaxis transducer	2.87	0.0000
PA14_46030	chemotaxis transducer	2.90	0.0000
PA14_26280	chemotaxis transducer	2.91	0.0000
PA14_20750	chemotaxis protein	3.35	0.0000
	DNA replication, recombination, modification and repair		
PA14_07530	DNA primase	2.04	0.0000
PA14_44610	recombination protein RecR	2.18	0.0000
PA14_44130	hypothetical protein	2.25	0.0000
PA14_59180	topoisomerase I - like protein	2.32	0.0000
PA14_17500	DNA mismatch repair protein MutS	2.46	0.0000
PA14_00110	DNA-3-methyladenine glycosidase I	2.55	0.0000
PA14_36910	ATP-dependent DNA ligase	2.56	0.0000
PA14_65130	replicative DNA helicase	2.82	0.0000
	Energy metabolism		
PA14_13030	CioA"	2.04	0.0000
PA14_20200	nitrous-oxide reductase	2.04	0.0000
PA14_39530	hydroxylase molybdopterin-containing subunit	2.04	0.0000
PA14_01300	cytochrome c oxidase subunit I	2.07	0.0001
PA14_06750	nitrite reductase	2.08	0.0000
PA14_49250	nitrate reductase catalytic subunit	2.17	0.0000
PA14_49270	cytochrome c-type protein NapC	2.20	0.0000
PA14_69110	Oxidoreductase	2.24	0.0000

PA14_17930	glycerol-3-phosphate dehydrogenase	2.36	0.0000
PA14_49220	ferredoxin protein NapF	2.37	0.0000
PA14_40730	ribosomal RNA large subunit methyltransferase N	2.42	0.0000
PA14_63580	nitrate-inducible formate dehydrogenase subunit beta	2.52	0.0000
PA14_30180	monomeric isocitrate dehydrogenase	2.58	0.0000
PA14_42120	hypothetical protein	2.58	0.0000
PA14_31770	Oxidoreductase	2.64	0.0000
PA14_68440	Oxidoreductase	2.84	0.0000
PA14_49230	NapD protein of periplasmic nitrate reductase	3.01	0.0000
PA14_01290	cytochrome c oxidase subunit II	3.04	0.0000
PA14_52380	cytochrome b561	3.08	0.0000
PA14_60490	cytochrome c	3.19	0.0000
PA14_49260	cytochrome c-type protein NapB precursor	3.28	0.0000
PA14_17490	ferredoxin I	5.20	0.0000
Fatty acid and phospholipid metabolism			
PA14_27160	acyl-CoA thioesterase	2.05	0.0000
PA14_25820	Lipoprotein	2.06	0.0001
PA14_13110	long-chain-fatty-acid--CoA ligase	2.12	0.0000
PA14_43680	3-hydroxydecanoyl-ACP dehydratase	2.13	0.0000
PA14_24880	Lipoprotein	2.17	0.0000
PA14_25640	glycerol-3-phosphate acyltransferase PlsX	2.22	0.0000
PA14_43420	acyl-CoA dehydrogenase	2.29	0.0000
PA14_36270	Dehydrogenase	2.33	0.0000
PA14_17880	acetyl-CoA acetyltransferase	2.33	0.0000
PA14_16560	Lipoprotein	2.33	0.0000
PA14_27910	hypothetical protein	2.46	0.0000
PA14_16610	hypothetical protein	2.54	0.0000
PA14_31760	phosphatidate cytidylyltransferase	2.55	0.0000
PA14_17850	enoyl-CoA hydratase	2.76	0.0000
PA14_17860	3-hydroxyacyl-CoA dehydrogenase	2.87	0.0000
PA14_16010	hypothetical protein	3.01	0.0000
PA14_19740	enoyl-CoA hydratase	3.30	0.0000
PA14_16640	Lipoprotein	3.81	0.0000
PA14_17820	hypothetical protein	3.86	0.0000
PA14_72900	Lipoprotein	4.24	0.0000
PA14_66350	acyl-CoA dehydrogenase	5.16	0.0000
PA14_31580	acyl-CoA dehydrogenase	6.41	0.0000
Hypothetical, unclassified, unknown			
PA14_36375	hypothetical protein	2.01	0.0000
PA14_30030	hypothetical protein	2.01	0.0003
PA14_53840	hypothetical protein	2.01	0.0007
PA14_07650	SpoVR family protein	2.01	0.0001
PA14_49290	hypothetical protein	2.02	0.0000

PA14_62240	hypothetical protein	2.03	0.0000
PA14_07630	hypothetical protein	2.03	0.0000
PA14_40330	hypothetical protein	2.04	0.0000
PA14_08330	hypothetical protein	2.04	0.0000
PA14_12700	hypothetical protein	2.04	0.0001
PA14_21670	hypothetical protein	2.05	0.0000
PA14_33200	hypothetical protein	2.05	0.0001
PA14_72350	hypothetical protein	2.05	0.0000
PA14_36790	hypothetical protein	2.06	0.0000
PA14_36530	hypothetical protein	2.06	0.0001
PA14_42150	hypothetical protein	2.07	0.0000
PA14_12620	hypothetical protein	2.07	0.0000
PA14_58620	hypothetical protein	2.08	0.0000
PA14_52440	hypothetical protein	2.08	0.0001
PA14_54260	hypothetical protein	2.09	0.0000
PA14_28230	hypothetical protein	2.09	0.0000
PA14_03420	hypothetical protein	2.09	0.0000
PA14_24260	hypothetical protein	2.09	0.0000
PA14_19750	hypothetical protein	2.10	0.0000
PA14_28140	hypothetical protein	2.10	0.0002
PA14_51850	hypothetical protein	2.10	0.0001
PA14_05775	hypothetical protein	2.11	0.0030
PA14_46080	hypothetical protein	2.13	0.0000
PA14_49320	hypothetical protein	2.13	0.0000
PA14_07660	hypothetical protein	2.13	0.0001
PA14_22160	hypothetical protein	2.13	0.0015
PA14_44170	hypothetical protein	2.14	0.0001
PA14_53690	hypothetical protein	2.14	0.0000
PA14_60520	hypothetical protein	2.15	0.0000
PA14_58260	hypothetical protein	2.15	0.0000
PA14_03166	hypothetical protein	2.16	0.0000
PA14_19330	hypothetical protein	2.16	0.0000
PA14_36560	hypothetical protein	2.16	0.0001
PA14_12560	hypothetical protein	2.17	0.0004
PA14_24560	hypothetical protein	2.17	0.0000
PA14_42160	hypothetical protein	2.17	0.0000
PA14_20460	hypothetical protein	2.18	0.0000
PA14_16340	hypothetical protein	2.18	0.0000
PA14_41990	hypothetical protein	2.19	0.0000
PA14_11670	hypothetical protein	2.19	0.0001
PA14_59490	hypothetical protein	2.19	0.0000
PA14_48930	coat protein A of bacteriophage Pf1	2.19	0.0000
PA14_49930	hypothetical protein	2.20	0.0000

PA14_46460	hypothetical protein	2.20	0.0002
PA14_36520	hypothetical protein	2.20	0.0000
PA14_32830	hypothetical protein	2.20	0.0000
PA14_36900	hypothetical protein	2.21	0.0000
PA14_53820	hypothetical protein	2.21	0.0000
PA14_58290	hypothetical protein	2.23	0.0000
PA14_51490	hypothetical protein	2.23	0.0000
PA14_59170	hypothetical protein	2.24	0.0001
PA14_46380	hypothetical protein	2.24	0.0000
PA14_03340	hypothetical protein	2.25	0.0000
PA14_34070	HsiB3	2.26	0.0000
PA14_19830	hypothetical protein	2.26	0.0000
PA14_36470	hypothetical protein	2.26	0.0000
PA14_50250	flagellar protein FliS	2.26	0.0000
PA14_08180	hypothetical protein	2.27	0.0001
PA14_20630	hypothetical protein	2.28	0.0000
PA14_03285	hypothetical protein	2.28	0.0000
PA14_28610	hypothetical protein	2.29	0.0000
PA14_47130	hypothetical protein	2.30	0.0000
PA14_35760	hypothetical protein	2.31	0.0000
PA14_58330	hypothetical protein	2.33	0.0000
PA14_28010	hypothetical protein	2.33	0.0000
PA14_53580	hypothetical protein	2.34	0.0000
PA14_52480	hypothetical protein	2.34	0.0000
PA14_49880	hypothetical protein	2.34	0.0000
PA14_40690	hypothetical protein	2.35	0.0000
PA14_00470	hypothetical protein	2.36	0.0000
PA14_59120	hypothetical protein	2.38	0.0000
PA14_52490	hypothetical protein	2.40	0.0000
PA14_59500	hypothetical protein	2.41	0.0000
PA14_42780	hypothetical protein	2.41	0.0000
PA14_24770	hypothetical protein	2.43	0.0000
PA14_28220	hypothetical protein	2.44	0.0000
PA14_12350	(dimethylallyl)adenosine tRNA methylthiotransferase	2.45	0.0000
PA14_27930	hypothetical protein	2.45	0.0000
PA14_43910	hypothetical protein	2.45	0.0000
PA14_65090	hypothetical protein	2.46	0.0000
PA14_28240	hypothetical protein	2.46	0.0000
PA14_72920	hypothetical protein	2.46	0.0000
PA14_03310	hypothetical protein	2.47	0.0000
PA14_53500	hypothetical protein	2.47	0.0000
PA14_53680	hypothetical protein	2.48	0.0000
PA14_62780	hypothetical protein	2.49	0.0000

PA14_61410	hypothetical protein	2.49	0.0000
PA14_00130	hypothetical protein	2.49	0.0000
PA14_44620	hypothetical protein	2.49	0.0000
PA14_43900	hypothetical protein	2.52	0.0001
PA14_28200	hypothetical protein	2.53	0.0000
PA14_05060	hypothetical protein	2.53	0.0000
PA14_57850	hypothetical protein	2.54	0.0000
PA14_05580	hypothetical protein	2.55	0.0000
PA14_59510	hypothetical protein	2.56	0.0000
PA14_16680	hypothetical protein	2.58	0.0000
PA14_59540	hypothetical protein	2.58	0.0000
PA14_50870	hypothetical protein	2.58	0.0000
PA14_62680	hypothetical protein	2.59	0.0000
PA14_44640	hypothetical protein	2.62	0.0000
PA14_28260	hypothetical protein	2.64	0.0000
PA14_41760	hypothetical protein	2.65	0.0000
PA14_28210	hypothetical protein	2.65	0.0000
PA14_13210	hypothetical protein	2.65	0.0000
PA14_49040	hypothetical protein	2.66	0.0000
PA14_21120	hypothetical protein	2.67	0.0000
PA14_52430	hypothetical protein	2.67	0.0000
PA14_32840	hypothetical protein	2.67	0.0000
PA14_46830	hypothetical protein	2.67	0.0000
PA14_63130	hypothetical protein	2.67	0.0000
PA14_41790	hypothetical protein	2.71	0.0000
PA14_54340	hypothetical protein	2.71	0.0000
PA14_19720	hypothetical protein	2.72	0.0000
PA14_53560	hypothetical protein	2.73	0.0000
PA14_53530	hypothetical protein	2.74	0.0000
PA14_54750	hypothetical protein	2.74	0.0000
PA14_47120	hypothetical protein	2.75	0.0000
PA14_59480	hypothetical protein	2.76	0.0000
PA14_68840	hypothetical protein	2.77	0.0000
PA14_41740	hypothetical protein	2.77	0.0000
PA14_59010	hypothetical protein	2.77	0.0000
PA14_54240	hypothetical protein	2.79	0.0000
PA14_27830	hypothetical protein	2.79	0.0000
PA14_52640	hypothetical protein	2.81	0.0000
PA14_00720	hypothetical protein	2.81	0.0000
PA14_72370	hypothetical protein	2.82	0.0000
PA14_29330	hypothetical protein	2.83	0.0000
PA14_28020	hypothetical protein	2.85	0.0000
PA14_46390	hypothetical protein	2.85	0.0000

PA14_19480	hypothetical protein	2.86	0.0000
PA14_35780	hypothetical protein	2.86	0.0000
PA14_59530	hypothetical protein	2.90	0.0000
PA14_31190	hypothetical protein	2.90	0.0000
PA14_07550	hypothetical protein	2.94	0.0000
PA14_31200	hypothetical protein	2.95	0.0000
PA14_49310	hypothetical protein	2.95	0.0000
PA14_11940	hypothetical protein	2.97	0.0000
PA14_01540	hypothetical protein	2.99	0.0000
PA14_47530	hypothetical protein	3.02	0.0000
PA14_17000	hypothetical protein	3.04	0.0000
PA14_21190	hypothetical protein	3.05	0.0000
PA14_36860	hypothetical protein	3.07	0.0000
PA14_62690	hypothetical protein	3.13	0.0000
PA14_47420	hypothetical protein	3.14	0.0000
PA14_72360	hypothetical protein	3.15	0.0000
PA14_59520	hypothetical protein	3.25	0.0000
PA14_00080	hypothetical protein	3.25	0.0000
PA14_50850	hypothetical protein	3.26	0.0000
PA14_14450	hypothetical protein	3.30	0.0000
PA14_00150	hypothetical protein	3.32	0.0000
PA14_41980	hypothetical protein	3.33	0.0000
PA14_53620	hypothetical protein	3.38	0.0000
PA14_24310	BNR/Asp-box repeat-containing protein	3.45	0.0000
PA14_06390	hypothetical protein	3.50	0.0000
PA14_53390	hypothetical protein	3.52	0.0000
PA14_12260	hypothetical protein	3.55	0.0000
PA14_03160	hypothetical protein	3.59	0.0000
PA14_50280	hypothetical protein	3.63	0.0000
PA14_20470	hypothetical protein	3.65	0.0000
PA14_40710	hypothetical protein	3.76	0.0000
PA14_54740	hypothetical protein	3.76	0.0000
PA14_61940	hypothetical protein	3.97	0.0000
PA14_20480	hypothetical protein	4.02	0.0000
PA14_50240	hypothetical protein	4.13	0.0000
PA14_08270	hypothetical protein	4.37	0.0000
PA14_59440	hypothetical protein	4.45	0.0000
PA14_07990	hypothetical protein	4.51	0.0000
PA14_36920	hypothetical protein	4.54	0.0000
PA14_08100	hypothetical protein	4.60	0.0000
PA14_16020	hypothetical protein	4.72	0.0000
PA14_11910	hypothetical protein	4.76	0.0000
PA14_08220	hypothetical protein	4.96	0.0000

PA14_62250	hypothetical protein	5.02	0.0000
PA14_72060	hypothetical protein	5.38	0.0000
PA14_08200	hypothetical protein	6.15	0.0000
PA14_08230	hypothetical protein	8.46	0.0000
Membrane proteins			
PA14_71590	hypothetical protein	2.05	0.0012
PA14_16630	outer membrane protein"	2.07	0.0002
PA14_17920	membrane protein GlpM	2.14	0.0000
PA14_11930	hypothetical protein	2.16	0.0000
PA14_05890	stomatin-like protein	2.31	0.0000
PA14_27070	hypothetical protein	2.42	0.0000
PA14_21840	hypothetical protein	2.49	0.0000
PA14_22350	acetate permease	2.50	0.0000
PA14_41750	hypothetical protein	2.56	0.0000
PA14_21130	outer membrane lipoprotein	2.68	0.0000
PA14_65040	hypothetical protein	2.91	0.0000
PA14_54520	porin	2.92	0.0000
PA14_17890	porin	3.00	0.0000
PA14_15100	hypothetical protein	3.09	0.0000
PA14_49050	hypothetical protein	3.13	0.0000
PA14_22340	hypothetical protein	3.23	0.0001
PA14_18720	OmpA family membrane protein	3.37	0.0000
PA14_42100	hypothetical protein	3.81	0.0000
Motility & Attachment			
PA14_11080	usher CupB3	2.05	0.0000
PA14_50450	flagellar hook protein FlgE	2.05	0.0002
PA14_50480	flagellar basal body rod protein FlgB	2.13	0.0000
PA14_11090	chaperone CupB4	2.26	0.0000
PA14_11060	fimbrial subunit hB1	2.29	0.0000
PA14_50470	flagellar basal body rod protein FlgC	2.50	0.0000
PA14_50460	flagellar basal body rod modification protein	2.71	0.0000
PA14_20740	flagellar basal body P-ring biosynthesis protein FlgA	3.17	0.0000
PA14_50290	flagellin type B	3.18	0.0000
PA14_50160	flagellar hook-basal body protein FliE	3.36	0.0000
Nucleotide biosynthesis and metabolism			
PA14_71530	formyltetrahydrofolate deformylase	2.10	0.0000
PA14_07700	diadenosine tetraphosphatase	2.30	0.0000
Protein secretion/export apparatus			
PA14_08695	preprotein translocase subunit SecE	2.20	0.0000
PA14_20720	hypothetical protein	2.50	0.0000
PA14_55940	hypothetical protein	2.77	0.0001
Putative enzymes			
PA14_35900	dehydrogenase	2.02	0.0004

PA14_01490	hemolysin	2.02	0.0003
PA14_11750	acetyltransferase	2.04	0.0004
PA14_58080	hypothetical protein	2.04	0.0001
PA14_61590	hypothetical protein	2.05	0.0000
PA14_46550	ribonuclease	2.09	0.0000
PA14_44510	hypothetical protein	2.12	0.0000
PA14_35980	acyl-CoA dehydrogenase	2.14	0.0001
PA14_27890	hypothetical protein	2.15	0.0000
PA14_68430	formate dehydrogenase accessory protein FdhD	2.20	0.0004
PA14_27990	sialidase	2.28	0.0000
PA14_46140	hypothetical protein	2.33	0.0000
PA14_49100	glutathione S-transferase	2.37	0.0000
PA14_10730	hypothetical protein	2.38	0.0000
PA14_17910	alpha/beta hydrolase	2.39	0.0000
PA14_52420	ribosomal protein S12 methylthiotransferase	2.39	0.0000
PA14_35670	glycosyl hydrolase	2.40	0.0000
PA14_17730	hypothetical protein	2.43	0.0000
PA14_44590	acyl-CoA dehydrogenase	2.49	0.0000
PA14_41780	hypothetical protein	2.53	0.0000
PA14_40890	short chain dehydrogenase	2.61	0.0000
PA14_49080	acyl-CoA dehydrogenase	2.74	0.0000
PA14_35880	gamma-aminobutyraldehyde dehydrogenase	2.83	0.0000
PA14_40180	oxidoreductase	2.85	0.0000
PA14_27880	hypothetical protein	2.90	0.0000
PA14_11810	aldehyde dehydrogenase	2.90	0.0001
PA14_53340	hypothetical protein	2.95	0.0000
PA14_53380	glycosyl transferase family protein	2.96	0.0000
PA14_04840	hypothetical protein	3.11	0.0000
PA14_68810	hypothetical protein	3.38	0.0000
PA14_35890	diaminobutyrate--2-oxoglutarate aminotransferase	3.53	0.0000
PA14_17810	acyl-CoA dehydrogenase	3.58	0.0000
PA14_53400	oxidoreductase	3.85	0.0000
PA14_40880	hypothetical protein	3.89	0.0000
PA14_19710	alpha/beta hydrolase	4.54	0.0000
PA14_08160	lytic enzyme	4.60	0.0000
PA14_46880	glutathione synthase	6.93	0.0000
PA14_19730	oxidoreductase	22.38	0.0000
Related to phage, transposon, or plasmid			
PA14_13890	integrase	2.07	0.0000
PA14_48990	hypothetical protein	2.17	0.0001
PA14_49000	hypothetical protein	2.20	0.0001
PA14_51620	transposase	2.25	0.0001
PA14_35820	cointegrate resolution protein S	2.64	0.0000

PA14_08300	phage-related protein"	3.10	0.0000
PA14_30930	TrbC-like protein	3.15	0.0000
PA14_30880	conjugal transfer protein TrbL	3.17	0.0000
PA14_08130	hypothetical protein	3.57	0.0000
PA14_08260	minor tail protein L	3.85	0.0000
PA14_08010	hypothetical protein	4.07	0.0000
PA14_08120	tail length determinator protein	4.22	0.0000
PA14_08050	tail fiber protein	4.33	0.0000
PA14_08280	bacteriophage protein	4.35	0.0000
PA14_08030	phage baseplate assembly protein	4.39	0.0000
PA14_08060	tail fiber assembly protein	4.53	0.0000
PA14_08090	phage tail tube protein	4.86	0.0000
PA14_08040	phage tail protein	4.96	0.0000
PA14_08070	phage tail sheath protein	5.21	0.0000
PA14_08250	hypothetical protein	5.22	0.0000
PA14_08150	phage late control gene D protein	5.38	0.0000
PA14_08140	hypothetical protein	5.52	0.0000
PA14_08210	hypothetical protein	6.05	0.0000
PA14_08240	hypothetical protein	6.21	0.0000
PA14_08020	bacteriophage protein	6.48	0.0000
	Secreted Factors (toxins, enzymes, alginate)		
PA14_69480	alginate biosynthesis protein AlgZ/FimS	2.75	0.0000
PA14_53360	hemolytic phospholipase C	2.91	0.0000
PA14_53370	phospholipase accessory protein PlcR	3.34	0.0000
	Transcription, RNA processing and degradation		
PA14_54330	ribonuclease III	2.06	0.0001
PA14_49060	hypothetical protein	2.18	0.0000
PA14_05560	ATP-dependent RNA helicase	2.37	0.0000
PA14_07970	hypothetical protein	2.52	0.0000
PA14_07730	dimethyladenosine transferase	2.68	0.0000
	Transcriptional regulators		
PA14_05960	cold-shock protein	2.01	0.0003
PA14_37660	transcriptional regulator	2.02	0.0000
PA14_25800	TetR family transcriptional regulator	2.08	0.0001
PA14_49110	transcriptional regulator	2.09	0.0000
PA14_54710	transcriptional regulator	2.09	0.0000
PA14_70560	LysR family transcriptional regulator	2.15	0.0000
PA14_73190	GlmR transcriptional regulator	2.19	0.0000
PA14_17900	transcriptional regulator MetR	2.20	0.0000
PA14_19850	transcriptional regulator	2.32	0.0000
PA14_51840	cold-shock protein	2.32	0.0000
PA14_36880	ompetence-damaged protein	2.38	0.0000
PA14_62490	suppressor protein DksA	2.41	0.0000

PA14_41800	transcriptional regulator	2.44	0.0000
PA14_19990	RNA polymerase ECF-subfamily sigma-70 factor	2.56	0.0000
PA14_46060	transcriptional regulator	2.61	0.0000
PA14_35540	transcriptional regulator BkdR	2.63	0.0000
PA14_20770	hypothetical protein	2.68	0.0000
PA14_28130	hypothetical protein	2.76	0.0000
PA14_26600	RNA polymerase sigma factor	2.77	0.0000
PA14_04930	RNA polymerase factor sigma-32	2.85	0.0000
PA14_54430	RNA polymerase sigma factor AlgU	3.16	0.0000
PA14_46330	transcriptional regulator	3.17	0.0000
PA14_30200	cold-shock protein CspD	3.37	0.0000
PA14_20730	hypothetical protein	3.91	0.0000
PA14_67550	transcriptional regulator	4.08	0.0000
PA14_53410	transcriptional regulator	4.45	0.0000
PA14_46850	transcriptional regulator	6.02	0.0000
Translation, post-translational modification, degradation			
PA14_30210	ATP-dependent Clp protease adaptor protein ClpS	2.03	0.0001
PA14_30230	ATP-dependent Clp protease"	2.14	0.0003
PA14_30260	arginyl-tRNA-protein transferase	2.30	0.0000
PA14_42130	hypothetical protein	2.38	0.0000
PA14_30240	translation initiation factor IF-1	2.39	0.0001
PA14_25630	50S ribosomal protein L32	2.43	0.0000
PA14_00100	glycyl-tRNA synthetase subunit alpha	2.53	0.0000
PA14_00090	glycyl-tRNA synthetase subunit beta	2.54	0.0000
PA14_07560	30S ribosomal protein S21	2.62	0.0000
PA14_60500	peptidyl-prolyl cis-trans isomerase FklB	2.82	0.0000
PA14_18650	hypothetical protein	2.86	0.0000
PA14_65150	50S ribosomal protein L9	2.96	0.0000
PA14_65320	tRNA delta(2)-isopentenylpyrophosphate transferase	2.99	0.0000
PA14_57470	hypothetical protein	4.40	0.0000
Transport of small molecules			
PA14_31970	CzcC family cobalt/zinc/cadmium efflux transporter outer membrane protein	2.01	0.0000
PA14_46950	ABC transporter ATP-binding protein	2.02	0.0000
PA14_01690	ABC transporter permease	2.04	0.0000
PA14_48680	hypothetical protein	2.05	0.0000
PA14_35860	amino acid permease	2.07	0.0001
PA14_35920	acetate permease	2.08	0.0000
PA14_53330	sulfate transport protein CysZ	2.08	0.0000
PA14_67300	ABC transporter substrate-binding protein	2.11	0.0000
PA14_07860	ABC transporter ATP-binding protein	2.12	0.0000
PA14_46010	ABC transporter ATP-binding protein	2.12	0.0000
PA14_01560	hypothetical protein	2.14	0.0000

PA14_49130	C4-dicarboxylate transporter DctA	2.16	0.0000
PA14_16880	ABC transporter permease	2.16	0.0000
PA14_52460	Mg transporter MgtE	2.18	0.0000
PA14_46800	hypothetical protein	2.20	0.0000
PA14_43400	potassium-transporting ATPase subunit A	2.22	0.0001
PA14_67280	ABC transporter permease	2.24	0.0000
PA14_54150	sodium/proline symporter PutP	2.27	0.0000
PA14_46910	ABC transporter substrate-binding protein	2.28	0.0000
PA14_52400	potassium uptake protein Kup	2.30	0.0000
PA14_54110	transporter	2.38	0.0000
PA14_04080	ABC transporter permease	2.38	0.0000
PA14_63050	hypothetical protein	2.40	0.0000
PA14_27840	hypothetical protein	2.42	0.0000
PA14_43405	potassium-transporting ATPase subunit F	2.57	0.0000
PA14_04090	ABC transporter substrate-binding protein	2.60	0.0000
PA14_72960	MFS dicarboxylate transporter	2.62	0.0000
PA14_73150	hypothetical protein	2.68	0.0000
PA14_07690	thiosulfate sulfurtransferase	2.78	0.0000
PA14_46100	transporter	2.85	0.0000
PA14_46110	sodium:solute symport protein	2.87	0.0000
PA14_48630	MFS transporter	2.88	0.0000
PA14_11790	amino acid transporter	2.99	0.0000
PA14_46930	ABC transporter permease	3.00	0.0000
PA14_31610	TerC family protein	3.01	0.0000
PA14_34960	glucose-sensitive porin	3.21	0.0000
PA14_43380	potassium-transporting ATPase subunit B	3.75	0.0000
PA14_47560	MFS transporter	3.92	0.0000
PA14_68800	hypothetical protein	3.94	0.0000
PA14_37380	flavin-binding monooxygenase	4.10	0.0000
PA14_43370	potassium-transporting ATPase subunit C	6.08	0.0000
	Two-component regulatory systems		
PA14_22370	hypothetical protein	2.05	0.0000
PA14_02260	two-component response regulator	2.08	0.0000
PA14_36420	sensor/response regulator hybrid	2.10	0.0000
PA14_63150	two-component response regulator	2.15	0.0000
PA14_62260	hypothetical protein	2.26	0.0000
PA14_06310	ACT domain-containing protein	2.34	0.0000
PA14_16350	two-component response regulator	2.34	0.0000
PA14_46370	two-component sensor	2.38	0.0000
PA14_30840	signal transduction histidine kinase	2.47	0.0000
PA14_07680	hypothetical protein	2.57	0.0000
PA14_63210	two-component response regulator	2.75	0.0000

Table S3: List of PA14 genes downregulated during confinement at hexadecane-water interface for 1h.

Gene		Fold Change	p value
	Adaptation, protection		
PA14_66460	hypothetical protein	-2.64	0.0000
PA14_06130	hypothetical protein	-2.42	0.0000
PA14_56530	hypothetical protein	-2.33	0.0000
PA14_49760	rhamnosyltransferase 2	-2.19	0.0000
	Amino acid biosynthesis and metabolism		
PA14_71650	aspartate ammonia-lyase	-2.60	0.0000
PA14_67150	oxidoreductase	-2.58	0.0000
PA14_23930	O-succinylhomoserine sulfhydrylase	-2.10	0.0000
PA14_15910	hypothetical protein	-2.07	0.0000
PA14_64850	ornithine cyclodeaminase	-2.02	0.0000
PA14_11400	riboflavin-specific deaminase/reductase	-2.06	0.0000
	Biosynthesis of cofactors, prosthetic groups and carriers		
PA14_21520	hypothetical protein	-4.18	0.0000
PA14_33520	thioesterase	-4.02	0.0000
PA14_64680	hypothetical protein	-2.88	0.0000
PA14_26460	cobalt-precorrin-6x reductase	-2.61	0.0000
PA14_47760	cobyrinic acid a"	-2.45	0.0000
PA14_26470	cobalt-precorrin-6A synthase	-2.33	0.0000
PA14_06570	dithiobiotin synthetase	-2.26	0.0000
PA14_47720	threonine-phosphate decarboxylase	-2.25	0.0000
PA14_68980	2-octaprenyl-6-methoxyphenyl hydroxylase	-2.17	0.0000
PA14_64940	hypothetical protein	-2.16	0.0000
PA14_30630	FAD-dependent monooxygenase	-2.16	0.0000
PA14_49820	cobalamin biosynthetic protein	-2.13	0.0000
PA14_39010	pyrroloquinoline quinone biosynthesis protein F	-2.12	0.0000
PA14_47670	nicotinate-nucleotide--dimethylbenzimidazole phosphoribosyltransferase	-2.10	0.0014
PA14_23880	folylpolyglutamate synthetase	-2.10	0.0000
PA14_26480	precorrin-6y-dependent methyltransferase CobL	-2.10	0.0000
PA14_64650	urease accessory protein UreE	-2.07	0.0019
PA14_13680	short chain dehydrogenase	-2.05	0.0001
PA14_47650	cobalamin synthase	-2.04	0.0004
	Carbon compound catabolism		
PA14_10230	2"	-2.67	0.0005
PA14_37770	hydrolase	-2.65	0.0000
PA14_10240	branched-chain alpha-keto acid dehydrogenase subunit E2	-2.50	0.0014

PA14_10260	dehydrogenase E1 component	-2.48	0.0004
PA14_34350	xylulose kinase	-2.31	0.0000
PA14_38550	maleylacetoacetate isomerase	-2.24	0.0000
PA14_34340	fructokinase	-2.21	0.0000
PA14_10270	hypothetical protein	-2.18	0.0003
PA14_38510	homogentisate 1"	-2.17	0.0000
PA14_39280	ribokinase	-2.02	0.0000
PA14_10250	acetoin catabolism protein AcoB	-2.01	0.0068
PA14_10590	2-oxo-hepta-3-ene-1"	-2.00	0.0004
	Cell wall / LPS / capsule		
PA14_35190	penicillin-binding protein 3A	-2.84	0.0000
PA14_73040	N-acetylmuramoyl-L-alanine amidase	-2.35	0.0000
PA14_11250	hypothetical protein	-2.31	0.0000
PA14_68210	dTDP-4-dehydrorhamnose 3"	-2.02	0.0000
PA14_68190	dTDP-4-dehydrorhamnose reductase	-2.02	0.0000
	Central intermediary metabolism		
PA14_38210	hypothetical protein	-2.63	0.0000
PA14_04580	dihydrofolate reductase	-2.40	0.0000
PA14_38200	thiamine pyrophosphate protein	-2.38	0.0000
PA14_37950	carbonate dehydratase	-2.20	0.0000
PA14_11260	epimerase	-2.09	0.0000
PA14_47970	hypothetical protein	-2.01	0.0000
	Chemotaxis		
PA14_48030	methyl-accepting chemotaxis transducer	-3.08	0.0000
PA14_64060	chemotaxis transducer	-2.04	0.0000
	DNA replication, recombination, modification and repair		
PA14_12630	ATP-dependent helicase	-2.26	0.0000
PA14_00230	Rossmann fold nucleotide-binding protein	-2.22	0.0005
	Energy metabolism		
PA14_63090	L-lactate dehydrogenase	-3.32	0.0000
PA14_22710	hypothetical protein	-3.02	0.0000
PA14_35150	alcohol dehydrogenase	-2.80	0.0000
PA14_64550	hypothetical protein	-2.25	0.0046
PA14_69140	CDP-6-deoxy-delta-3"	-2.15	0.0000
PA14_06770	regulatory protein NirQ	-2.13	0.0000
PA14_65940	oxidoreductase	-2.07	0.0000
PA14_10500	cbb3-type cytochrome c oxidase subunit I	-2.04	0.0005
	Fatty acid and phospholipid metabolism		
PA14_39060	lipoprotein	-3.21	0.0000
PA14_60690	lipoprotein	-3.16	0.0002
PA14_71110	lipolytic protein	-2.31	0.0019
PA14_56410	phospholipase	-2.18	0.0000
PA14_05860	hypothetical protein	-2.17	0.0001

PA14_43600	hypothetical protein	-2.15	0.0000
PA14_26690	enoyl-CoA hydratase/isomerase	-2.05	0.0000
PA14_34490	hypothetical protein	-2.04	0.0012
PA14_38630	acetyl-CoA acetyltransferase	-2.00	0.0000
	Hypothetical, unclassified, unknown		
PA14_28410	hypothetical protein	-4.72	0.0000
PA14_33830	hypothetical protein	-4.30	0.0000
PA14_10490	hypothetical protein	-3.67	0.0000
PA14_11330	hypothetical protein	-3.56	0.0000
PA14_03090	hypothetical protein	-3.46	0.0000
PA14_09350	hypothetical protein	-3.19	0.0000
PA14_33570	hypothetical protein	-3.12	0.0000
PA14_21530	ankyrin domain-containing protein	-3.09	0.0000
PA14_48410	hypothetical protein	-2.99	0.0000
PA14_02960	hypothetical protein	-2.79	0.0000
PA14_43520	hypothetical protein	-2.78	0.0000
PA14_40280	hypothetical protein	-2.73	0.0000
PA14_68720	hypothetical protein	-2.71	0.0000
PA14_11170	hypothetical protein	-2.71	0.0000
PA14_21560	hypothetical protein	-2.66	0.0000
PA14_24150	hypothetical protein	-2.65	0.0000
PA14_56550	hypothetical protein	-2.63	0.0000
PA14_26990	hypothetical protein	-2.58	0.0000
PA14_42410	hypothetical protein	-2.56	0.0002
PA14_72500	hypothetical protein	-2.55	0.0000
PA14_26165	hypothetical protein	-2.54	0.0000
PA14_44990	hypothetical protein	-2.51	0.0000
PA14_21260	hypothetical protein	-2.50	0.0003
PA14_71430	hypothetical protein	-2.46	0.0000
PA14_64530	hypothetical protein	-2.42	0.0022
PA14_32290	hypothetical protein	-2.40	0.0000
PA14_38270	hypothetical protein	-2.39	0.0000
PA14_10020	hypothetical protein	-2.37	0.0000
PA14_39260	hypothetical protein	-2.35	0.0000
PA14_60480	hypothetical protein	-2.35	0.0000
PA14_33110	hypothetical protein	-2.35	0.0001
PA14_10220	SH3 domain-containing protein	-2.33	0.0006
PA14_38260	hypothetical protein	-2.32	0.0000
PA14_21510	hypothetical protein	-2.30	0.0000
PA14_33880	hypothetical protein	-2.30	0.0000
PA14_39070	hypothetical protein	-2.28	0.0000
PA14_58540	hypothetical protein	-2.28	0.0000
PA14_33250	hypothetical protein	-2.26	0.0001

PA14_39660	hypothetical protein	-2.23	0.0001
PA14_52110	hypothetical protein	-2.21	0.0000
PA14_42380	hypothetical protein	-2.20	0.0000
PA14_17650	hypothetical protein	-2.19	0.0001
PA14_31360	hypothetical protein	-2.15	0.0000
PA14_67620	hypothetical protein	-2.13	0.0001
PA14_24240	hypothetical protein	-2.12	0.0000
PA14_62640	hypothetical protein	-2.12	0.0000
PA14_42510	hypothetical protein	-2.10	0.0080
PA14_52010	DNA replication initiation factor	-2.10	0.0000
PA14_17580	hypothetical protein	-2.09	0.0000
PA14_64560	hypothetical protein	-2.09	0.0094
PA14_04520	hypothetical protein	-2.08	0.0000
PA14_52940	hypothetical protein	-2.06	0.0000
PA14_71380	hypothetical protein	-2.05	0.0001
PA14_16980	hypothetical protein	-2.05	0.0001
PA14_24410	hypothetical protein	-2.04	0.0094
PA14_33590	hypothetical protein	-2.04	0.0000
PA14_41030	hypothetical protein	-2.04	0.0014
PA14_60560	hypothetical protein	-2.04	0.0000
PA14_27490	hypothetical protein	-2.02	0.0001
PA14_48400	hypothetical protein	-2.01	0.0012
	Membrane proteins		
PA14_32720	transmembrane sensor	-3.36	0.0000
PA14_64690	transmembrane sensor	-3.29	0.0000
PA14_19390	hypothetical protein	-2.75	0.0000
PA14_09540	hypothetical protein	-2.73	0.0000
PA14_39810	transmembrane sensor	-2.63	0.0000
PA14_09500	outer membrane protein	-2.57	0.0000
PA14_69090	hypothetical protein	-2.48	0.0000
PA14_28980	Fe2+-dicitrate sensor	-2.47	0.0001
PA14_13450	transmembrane sensor	-2.45	0.0000
PA14_29660	hypothetical protein	-2.33	0.0000
PA14_22320	hypothetical protein	-2.17	0.0000
PA14_58500	hypothetical protein	-2.15	0.0000
PA14_26360	ABC transporter permease	-2.12	0.0001
PA14_27270	hypothetical protein	-2.07	0.0000
PA14_10680	hypothetical protein	-2.03	0.0000
PA14_22660	hypothetical protein	-2.03	0.0000
	Motility & Attachment		
PA14_50110	flagellar assembly protein H	-2.57	0.0000
PA14_33530	hypothetical protein	-2.47	0.0000
PA14_16440	hypothetical protein	-2.19	0.0000

Nucleotide biosynthesis and metabolism			
PA14_00800	hypothetical protein	-2.15	0.0000
PA14_73070	dihydroorotase	-2.14	0.0000
PA14_32340	deaminase	-2.03	0.0000
PA14_64930	hypothetical protein	-2.01	0.0000
Protein secretion/export apparatus			
PA14_10350	secretion protein	-2.73	0.0000
PA14_42360	type III export apparatus protein	-2.44	0.0008
PA14_10340	toxin transporter	-2.36	0.0000
PA14_10330	outer membrane protein	-2.33	0.0000
PA14_55430	HxcS	-2.20	0.0000
PA14_20050	outer membrane protein	-2.09	0.0000
PA14_55470	HxcY	-2.08	0.0003
PA14_23980	secretion protein XcpP	-2.05	0.0000
PA14_55530	HxcW	-2.02	0.0002
PA14_20040	metalloprotease secretion protein	-2.00	0.0001
Putative enzymes			
PA14_68955	2-octaprenyl-3-methyl-6-methoxy-1"	-2.68	0.0000
PA14_64540	hypothetical protein	-2.63	0.0005
PA14_35200	acetyltransferase	-2.42	0.0000
PA14_10280	short-chain dehydrogenase	-2.39	0.0005
PA14_31840	hypothetical protein	-2.33	0.0000
PA14_01750	hydroxydechloroatrazine ethylaminohydrolase	-2.32	0.0000
PA14_47860	oxidoreductase	-2.28	0.0001
PA14_10130	short chain dehydrogenase	-2.27	0.0000
PA14_53300	alkyl hydroperoxide reductase	-2.23	0.0000
PA14_41400	UDP-2"	-2.21	0.0000
PA14_57240	hypothetical protein	-2.19	0.0000
PA14_37745	carbamoyl transferase	-2.19	0.0000
PA14_09490	phenazine-specific methyltransferase	-2.19	0.0000
PA14_63470	methyltransferase	-2.18	0.0000
PA14_10900	alcohol dehydrogenase	-2.16	0.0000
PA14_39250	double-glycine peptidase	-2.14	0.0000
PA14_23650	short chain dehydrogenase	-2.11	0.0004
PA14_58515	hypothetical protein	-2.10	0.0000
PA14_43540	hydrolase	-2.10	0.0000
PA14_31370	hypothetical protein	-2.08	0.0006
PA14_09950	oxidoreductase	-2.06	0.0001
PA14_11130	short chain dehydrogenase	-2.05	0.0000
PA14_32590	thiol:disulfide interchange protein	-2.02	0.0001
PA14_11020	3-ketoacyl-ACP reductase	-2.01	0.0001
PA14_49690	oxidoreductase	-2.00	0.0000
Secreted Factors (toxins, enzymes, alginate)			

PA14_09450	phenazine biosynthesis protein PhzD	-4.21	0.0000
PA14_48040	alkaline proteinase inhibitor AprI	-2.22	0.0000
PA14_35390	pyoverdine biosynthesis protein PvcD	-2.21	0.0020
PA14_35400	pyoverdine biosynthesis protein PvcC	-2.08	0.0001
PA14_09410	pyrodoxamine 5'-phosphate oxidase	-2.06	0.0000
	Transcriptional regulators		
PA14_28420	LysR family transcriptional regulator	-3.24	0.0000
PA14_35170	redox-sensing activator of soxS	-3.09	0.0000
PA14_48830	transcriptional regulator	-2.89	0.0000
PA14_10190	transcriptional regulator	-2.79	0.0000
PA14_66490	LysR family transcriptional regulator	-2.73	0.0000
PA14_47610	transcriptional regulator	-2.63	0.0000
PA14_01840	RNA polymerase ECF-subfamily sigma-70 factor	-2.51	0.0000
PA14_16380	LysR family transcriptional regulator	-2.49	0.0000
PA14_38500	IclR family transcriptional regulator	-2.47	0.0000
PA14_48810	NAD-dependent deacetylase	-2.45	0.0000
PA14_33840	transcriptional regulator	-2.44	0.0000
PA14_15150	transcriptional regulator	-2.42	0.0000
PA14_02910	IclR family transcriptional regulator	-2.37	0.0000
PA14_24140	AraC family transcriptional regulator	-2.37	0.0000
PA14_65950	transcriptional regulator	-2.33	0.0003
PA14_43580	transcriptional regulator	-2.33	0.0000
PA14_56070	transcriptional regulator MvaT''	-2.32	0.0000
PA14_48390	AraC family transcriptional regulator	-2.26	0.0000
PA14_54010	transcriptional regulator	-2.26	0.0000
PA14_34690	LysR family transcriptional regulator	-2.22	0.0000
PA14_10010	transcriptional regulator	-2.19	0.0000
PA14_67170	LysR family transcriptional regulator	-2.18	0.0000
PA14_23730	LysR family transcriptional regulator	-2.18	0.0000
PA14_48420	transcriptional regulator	-2.14	0.0000
PA14_09260	transcriptional regulator PchR	-2.14	0.0000
PA14_60810	transcriptional regulator NfxB	-2.13	0.0000
PA14_06180	RNA polymerase sigma factor	-2.13	0.0000
PA14_13470	AraC family transcriptional regulator	-2.08	0.0000
PA14_29440	LysR family transcriptional regulator	-2.07	0.0000
PA14_32940	transcriptional regulator	-2.05	0.0000
PA14_11180	transcriptional regulator	-2.05	0.0000
PA14_71640	LysR family transcriptional regulator	-2.04	0.0000
PA14_02870	transcriptional regulator	-2.02	0.0001
PA14_29620	anaerobic nitric oxide reductase transcriptional regulator	-2.01	0.0000
PA14_30450	LysR family transcriptional regulator	-2.00	0.0000
	Translation, post-translational modification, degradation		

PA14_34850	tRNA synthase	-2.15	0.0000
PA14_17440	tRNA pseudouridine synthase D	-2.12	0.0001
PA14_08890	30S ribosomal protein S19	-2.05	0.0000
PA14_00240	hypothetical protein	-2.02	0.0000
	Transport of small molecules		
PA14_03080	acetyltransferase	-3.40	0.0000
PA14_10160	ferric enterobactin transport protein FepD	-3.38	0.0000
PA14_37420	transmembrane sensor protein	-2.95	0.0000
PA14_33550	ABC transporter ATP-binding protein	-2.93	0.0000
PA14_10140	ferric enterobactin transport protein FepG	-2.88	0.0000
PA14_10180	ferric enterobactin transport protein FepC	-2.86	0.0000
PA14_22650	ABC transporter	-2.74	0.0000
PA14_40270	cation transporter	-2.70	0.0000
PA14_00860	ABC transporter ATP-binding protein	-2.69	0.0005
PA14_37290	hypothetical protein	-2.65	0.0000
PA14_33750	outer membrane protein	-2.65	0.0000
PA14_10470	MFS transporter	-2.64	0.0000
PA14_09530	RND efflux membrane fusion protein	-2.54	0.0000
PA14_60780	ABC transporter permease	-2.53	0.0000
PA14_37310	hypothetical protein	-2.52	0.0000
PA14_01860	transmembrane sensor	-2.43	0.0000
PA14_09520	RND efflux transporter	-2.32	0.0000
PA14_57990	hypothetical protein	-2.27	0.0000
PA14_15790	phosphoenolpyruvate-protein phosphotransferase	-2.25	0.0000
PA14_47900	MFS transporter	-2.22	0.0000
PA14_33540	ABC transporter permease	-2.21	0.0000
PA14_34330	hypothetical protein	-2.19	0.0000
PA14_03550	MFS transporter	-2.18	0.0000
PA14_47640	major facilitator transporter	-2.17	0.0000
PA14_65870	hypothetical protein	-2.16	0.0000
PA14_10170	iron-enterobactin transporter periplasmic binding protein	-2.14	0.0000
PA14_38730	major facilitator superfamily transporter	-2.13	0.0000
PA14_37840	ABC transporter ATP-binding protein	-2.12	0.0001
PA14_69340	ABC transporter ATP-binding protein	-2.11	0.0004
PA14_70120	MFS transporter	-2.10	0.0000
PA14_33760	ABC transporter ATP-binding protein/permease	-2.08	0.0000
PA14_60790	ABC transporter ATP-binding protein	-2.07	0.0000
PA14_38220	hypothetical protein	-2.06	0.0000
PA14_26390	hypothetical protein	-2.05	0.0000
PA14_43660	hypothetical protein	-2.05	0.0004
PA14_09380	transporter	-2.01	0.0000
PA14_34500	ABC transporter ATP-binding protein	-2.01	0.0061

	Two-component regulatory systems		
PA14_06170	transmembrane sensor	-2.66	0.0000
PA14_47390	transmembrane sensor	-2.56	0.0000
PA14_29375	possible C-terminal fragment of two-component response regulator PfeR	-2.51	0.0000
PA14_29360	two-component sensor PfeS	-2.44	0.0000
PA14_64410	hypothetical protein	-2.27	0.0000
PA14_65860	two-component sensor	-2.17	0.0000
PA14_27570	GAF domain-containing protein	-2.06	0.0000

Table S4: List of PAO1 genes upregulated during confinement at hexadecane-water interface for 1h.

Gene	**Description**	**Fold Change**
	Adaptation, Protection	
motA	chemotaxis protein MotA	2.93
PA3818	extragenic suppressor protein SuhB	2.66
	Biosynthesis of cofactors, prosthetic groups and carriers	
lipA	lipoate synthase	4.68
ureE	urease accessory protein UreE	3.63
ureG	urease accessory protein UreG	2.52
	Carbon compound catabolism	
alkB2	alkane-1-monooxygenase 2	11.32
PA2097	probable flavin-binding monooxygenase	2.67
	Cell wall / LPS / capsule	
rhlG	beta-ketoacyl reductase	2.62
bacA	bacitracin resistance protein	2.33
algD	GDP-mannose 6-dehydrogenase AlgD	2.08
	Chemotaxis	
PA1611	hybrid sensor kinase	4.42
	Energy metabolism	
fdxA	ferredoxin I	2.96
	Hypothetical, unclassified, unknown	
PA4682	hypothetical protein	18.36
PA0830	hypothetical protein	16.88
PA1542	hypothetical protein	9.57
PA3323	conserved hypothetical protein	8.80
PA1096	hypothetical protein	5.85
PA1761	hypothetical protein	5.13
PA3740	hypothetical protein	5.00
PA3741	hypothetical protein	4.72
PA1451	conserved hypothetical protein	4.49
PA5424	conserved hypothetical protein	4.41
PA1366	hypothetical protein	4.38

PA4703	hypothetical protein	4.28
PA4360a	hypothetical protein	4.19
PA4337	hypothetical protein	4.14
PA1190	conserved hypothetical protein	4.06
PA1786	NasS	4.02
PA0738	conserved hypothetical protein	3.99
PA4015	conserved hypothetical protein	3.95
PA2174	hypothetical protein	3.87
PA3767	conserved hypothetical protein	3.84
PA5286	conserved hypothetical protein	3.83
PA0311	hypothetical protein	3.79
PA5526	hypothetical protein	3.76
PA1612	hypothetical protein	3.74
PA3177	hypothetical protein	3.73
PA0490	hypothetical protein	3.65
PA0628	conserved hypothetical protein	3.65
PA0310	hypothetical protein	3.61
PA0621	conserved hypothetical protein	3.61
PA3954	hypothetical protein	3.51
PA0274	hypothetical protein	3.50
PA2137	hypothetical protein	3.47
PA3661	hypothetical protein	3.45
PA4437	hypothetical protein	3.34
PA1762	hypothetical protein	3.26
PA1466	hypothetical protein	3.11
PA4630	hypothetical protein	3.11
PA0850	hypothetical protein	3.08
PA1471	hypothetical protein	3.06
PA4691	hypothetical protein	3.01
PA1965	hypothetical protein	2.83
PA2959	conserved hypothetical protein	2.74
PA1383	hypothetical protein	2.70
PA0911	hypothetical protein	2.67
PA0042	hypothetical protein	2.65
PA3772	hypothetical protein	2.61
PA3012	hypothetical protein	2.56
PA4454	conserved hypothetical protein	2.55
PA3009	hypothetical protein	2.50
PA2173a	hypothetical protein	2.47
PA2075	hypothetical protein	2.45
PA1467	hypothetical protein	2.36
PA0012	hypothetical protein	2.36
PA3445	conserved hypothetical protein	2.33
PA4712	hypothetical protein	2.22
PA1298	conserved hypothetical protein	2.18
PA3762	hypothetical protein	2.15

PA4641	still frameshift hypothetical protein	2.96
	Membrane proteins	
PA3428	hypothetical protein	3.53
PA3403	hypothetical protein	3.28
PA3403a	hypothetical protein	2.94
PA1305	hypothetical protein	2.40
PA1591	hypothetical protein	2.33
PA1231	conserved hypothetical protein	2.27
	Motility & Attachment	
fliE	flagellar hook-basal body complex protein FliE	8.43
cupB4	chaperone CupB4	2.75
fimT	type 4 fimbrial biogenesis protein FimT	2.37
	Nucleotide biosynthesis and metabolism	
cysC	adenosine 5'-phosphosulfate (APS) kinase	2.23
rocR	RocR	2.15
	Putative enzymes	
PA1538	probable flavin-containing monooxygenase	16.09
PA3427	probable short-chain dehydrogenases	15.62
PA2550	probable acyl-CoA dehydrogenase	14.44
PA3277	probable short-chain dehydrogenase	8.59
PA1648	probable oxidoreductase	6.25
PA0840	probable oxidoreductase	5.88
PA3430	probable aldolase	4.21
PA0829	probable hydrolase	3.60
PA2142a	probable short-chain dehydrogenase	3.41
PA1185	probable glutathione S-transferase	2.22
	Related to phage, transposon, or plasmid	
PA0629	conserved hypothetical protein	3.39
PA0620	probable bacteriophage protein	3.25
PA0640	probable bacteriophage protein	3.20
PA0625	hypothetical protein	3.01
PA0627	hypothetical protein	2.96
PA0634	hypothetical protein	2.95
PA0639	conserved hypothetical protein	2.90
PA0644	hypothetical protein	2.90
PA0633	hypothetical protein	2.88
PA0624	hypothetical protein	2.72
PA0637	conserved hypothetical protein	2.71
PA0643	hypothetical protein	2.66
PA0617	probable bacteriophage protein	2.57
PA0645	hypothetical protein	2.57
PA0978	conserved hypothetical protein	2.50
PA0626	hypothetical protein	2.47
PA0631	hypothetical protein	2.35
	Transcriptional regulators	
PA0839	probable transcriptional regulator	5.78

flgM	FlgM	4.58
PA2354	probable transcriptional regulator	3.24
PA4784	probable transcriptional regulator	3.16
mexR	multidrug resistance operon repressor MexR	2.84
PA5116	probable transcriptional regulator	2.48
PA3714	probable two-component response regulator	2.34
PA2376	probable transcriptional regulator	2.31
	Transport of small molecules	
PA2073	probable transporter (membrane subunit)	4.65
PA3597	probable amino acid permease	2.26

Table S5: List of PAO1 genes downregulated during confinement at hexadecane-water interface for 1h.

Gene	Description	Fold Change
	Amino acid biosynthesis and metabolism	
gpuA	3-guanidinopropionase	-2.28
	Biosynthesis of cofactors, prosthetic groups and carriers	
pqqF	pyrroloquinoline quinone biosynthesis protein F	-2.15
	Carbon compound catabolism	
mdcE	malonate decarboxylase gamma subunit	-2.43
mdcC	malonate decarboxylase delta subunit	-2.32
	Hypothetical, unclassified, unknown	
PA3520	hypothetical protein	-3.23
PA4220	hypothetical protein	-2.89
PA3207	hypothetical protein	-2.56
PA2330	hypothetical protein	-2.54
PA2406	hypothetical protein	-2.35
PA4908	hypothetical protein	-2.25
PA3332	conserved hypothetical protein	-2.21
PA1218	hypothetical protein	-2.18
	Membrane proteins	
PA3432	hypothetical protein	-2.07
	Putative enzymes	
PA0883	probable acyl-CoA lyase beta chain	-2.94
PA3330	probable short chain dehydrogenase	-2.55
PA2263	probable 2-hydroxyacid dehydrogenase	-2.25
atuE	putative isohexenylglutaconyl-CoA hydratase	-2.17
	Secreted Factors (toxins, enzymes, alginate)	
phzC2	phenazine biosynthesis protein PhzC	-2.99
	Transcriptional regulators	
PA3574a	NalD	-3.63
PA2010	probable transcriptional regulator	-2.33
	Transport of small molecules	
PA3920	probable metal transporting P-type ATPase	-4.04

References

1 Sidorenko, J., Jatsenko, T. & Kivisaar, M. Ongoing evolution of Pseudomonas aeruginosa PAO1 sublines complicates studies of DNA damage repair and tolerance. *Mutation Research/Fundamental and Molecular Mechanisms of Mutagenesis* **797**, 26-37 (2017).

2 Jacobs, M. A. *et al.* Comprehensive transposon mutant library of Pseudomonas aeruginosa. *Proceedings of the National Academy of Sciences of the United States of America* **100**, 14339-14344, doi:10.1073/pnas.2036282100 (2003).

3 Luong, P. M. *et al.* Emergence of the P2 phenotype in Pseudomonas aeruginosa PAO1 strains involves various mutations in mexT or mexF. *Journal of bacteriology* **196**, 504-513 (2014).
